# Nucleotide diversity of functionally different groups of immune response genes in Old World camels based on newly annotated and reference-guided assemblies

**DOI:** 10.1186/s12864-020-06990-4

**Published:** 2020-09-03

**Authors:** Sara Lado, Jean P. Elbers, Mark F. Rogers, José Melo-Ferreira, Adiya Yadamsuren, Jukka Corander, Petr Horin, Pamela A. Burger

**Affiliations:** 1 Department of Interdisciplinary Life Sciences, Research Institute of Wildlife Ecology, Vetmeduni Vienna, Vienna, Austria; 2 Intelligent Systems Laboratory, University of Bristol, Bristol, UK; 3 CIBIO, Centro de Investigação em Biodiversidade e Recursos Genéticos, InBIO Laboratório Associado, Universidade do Porto, Vairão, Portugal; 4 Departamento de Biologia, Faculdade de Ciências da Universidade do Porto, Porto, Portugal; 5 Wild Camel Protection Foundation Mongolia, Jukov avenue, Bayanzurh District, Ulaanbaatar, 13343 Mongolia; 6 Wellcome Sanger Institute, Hinxton, UK; 7 Department of Mathematics and Statistics, Helsinki Institute for Information Technology, University of Helsinki, FIN-00014 Helsinki, Finland; 8 Department of Biostatistics, University of Oslo, N-0317 Oslo, Norway; 9 Department of Animal Genetics, Veterinary and Pharmaceutical University, Brno, Czech Republic; 10 Ceitec VFU, RG Animal Immunogenomics, Brno, Czech Republic

**Keywords:** Chromosome mapping, Chromosome conformation capture, Dromedary, Genome assembly, Scaffolding, Genome annotation, Immune response genes, Genetic diversity

## Abstract

**Background:**

Immune-response (IR) genes have an important role in the defense against highly variable pathogens, and therefore, diversity in these genomic regions is essential for species’ survival and adaptation. Although current genome assemblies from Old World camelids are very useful for investigating genome-wide diversity, demography and population structure, they have inconsistencies and gaps that limit analyses at local genomic scales. Improved and more accurate genome assemblies and annotations are needed to study complex genomic regions like adaptive and innate IR genes.

**Results:**

In this work, we improved the genome assemblies of the three Old World camel species – domestic dromedary and Bactrian camel, and the two-humped wild camel – via different computational methods. The newly annotated dromedary genome assembly CamDro3 served as reference to scaffold the NCBI RefSeq genomes of domestic Bactrian and wild camels. These upgraded assemblies were then used to assess nucleotide diversity of IR genes within and between species, and to compare the diversity found in immune genes and the rest of the genes in the genome. We detected differences in the nucleotide diversity among the three Old World camelid species and between IR gene groups, i.e., innate versus adaptive. Among the three species, domestic Bactrian camels showed the highest mean nucleotide diversity. Among the functionally different IR gene groups, the highest mean nucleotide diversity was observed in the major histocompatibility complex.

**Conclusions:**

The new camel genome assemblies were greatly improved in terms of contiguity and increased size with fewer scaffolds, which is of general value for the scientific community. This allowed us to perform in-depth studies on genetic diversity in immunity-related regions of the genome. Our results suggest that differences of diversity across classes of genes appear compatible with a combined role of population history and differential exposures to pathogens, and consequent different selective pressures.

## Background

Accurate genome assemblies provide an invaluable basis to assess genetic variation throughout the genome of species, to detect structural variants and to decipher complex genomic regions such as immune-response (IR) genes. Maintaining high genetic diversity in a population is important to reduce the spread of diseases, allowing rapid adequate immune responses and limiting, e.g., parasite evolution (see [1]). Even though demographic changes in general may cause important loss of genetic diversity, and particularly during domestication, due to intensive selection and potential inbreeding in many genomic regions [2], in other regions such as IR genes the genetic diversity can be conserved due to selective pressures of pathogens [3].

Old World camels (Artiodactyla, Tylopoda, Camelidae, Camelini) – the domesticated one-humped dromedaries (*Camelus dromedarius*) and two-humped Bactrian camels (*Camelus bactrianus*), as well as the critically endangered two-humped wild camels (*Camelus ferus*) – are valuable species not only for their production traits (e.g., meat, milk or wool), but for their power (e.g., riding or packing). Moreover, they are ungulate species with unique adaptations to diverse and extreme environments. Consequently, as they are in contact with different pathogenic pressures on different environments, there is great interest in understanding the general diversity in the part of the genome encoding their immune system. Previous research on immunogenome diversity in Old World camels focused mainly on the MHC genes (e.g.*,* [4]), as due to its critical importance for individual survival, the MHC complex is the most intensively studied part of the vertebrate immunogenome [5]. MHC genes, however, account only for part of the genetic variability underlying resistance to infectious pathogens [6, 7]. A broader approach is required to capture the overall genetic diversity of the immune system and to understand its role in response to pathogens. On these grounds, high-quality genome assemblies are needed. Previous studies [8–12] developed high quality genome assemblies for the three Old World camel species. Although very useful for broad inferences of genome-wide diversity or demographic histories, an improved version of these assemblies is needed to allow more detailed studies of the diversity in parts of the genome, such as IR genes. Access to different computational methods allows overcoming previous genome assemblies´ limitations.

In this work, we describe our computational efforts to generate improved Old World camelid genome assemblies, and we present versions CamDro3, CamBac2 and CamFer2, for dromedaries, Bactrian camels and wild camels, respectively. Our goal was not only to provide novel assemblies for genomic analysis in camels, but also to take advantage of the upgraded genome assemblies to assess the genetic diversity in different groups of immune genes, and compare them among species and to the rest of the intra-genic genomic diversity.

## Results

### Improved *Camelus dromedarius* genome assembly

Despite the utility of the CamDro1 and CamDro2 assemblies, inconsistencies and gaps can limit analyses at various genomic scales. By using different bioinformatic methods, we were able to upgrade the available genome assemblies to CamDro3, which is more accurate, contiguous and show fewer scaffolds of increased size when compared to the previous ones. CamDro3 consistently had higher RNA-Seq read mapping rates than CamDro2, and these two assemblies had much higher mapping rates than the other assemblies (Supplemental Fig. 1). After CamDro3 and CamDro2, the assembly with the third highest mapping rates varied depending on the tissue and season analyzed, but *B. taurus* consistently had the lowest mapping rates. We were able to assign at least one super-scaffold to each of the 37 chromosomes except the Y chromosome as the dromedary used in CamDro1, CamDro2, and CamDro3 was female. Chromosomes are denoted by numbers 1–36 and X in the CamDro3 assembly. There were 113,944,958 bases in scaffolds not assigned to chromosomes (5.25% of the 2,169,346,739 base assembly).

**Fig. 1:**
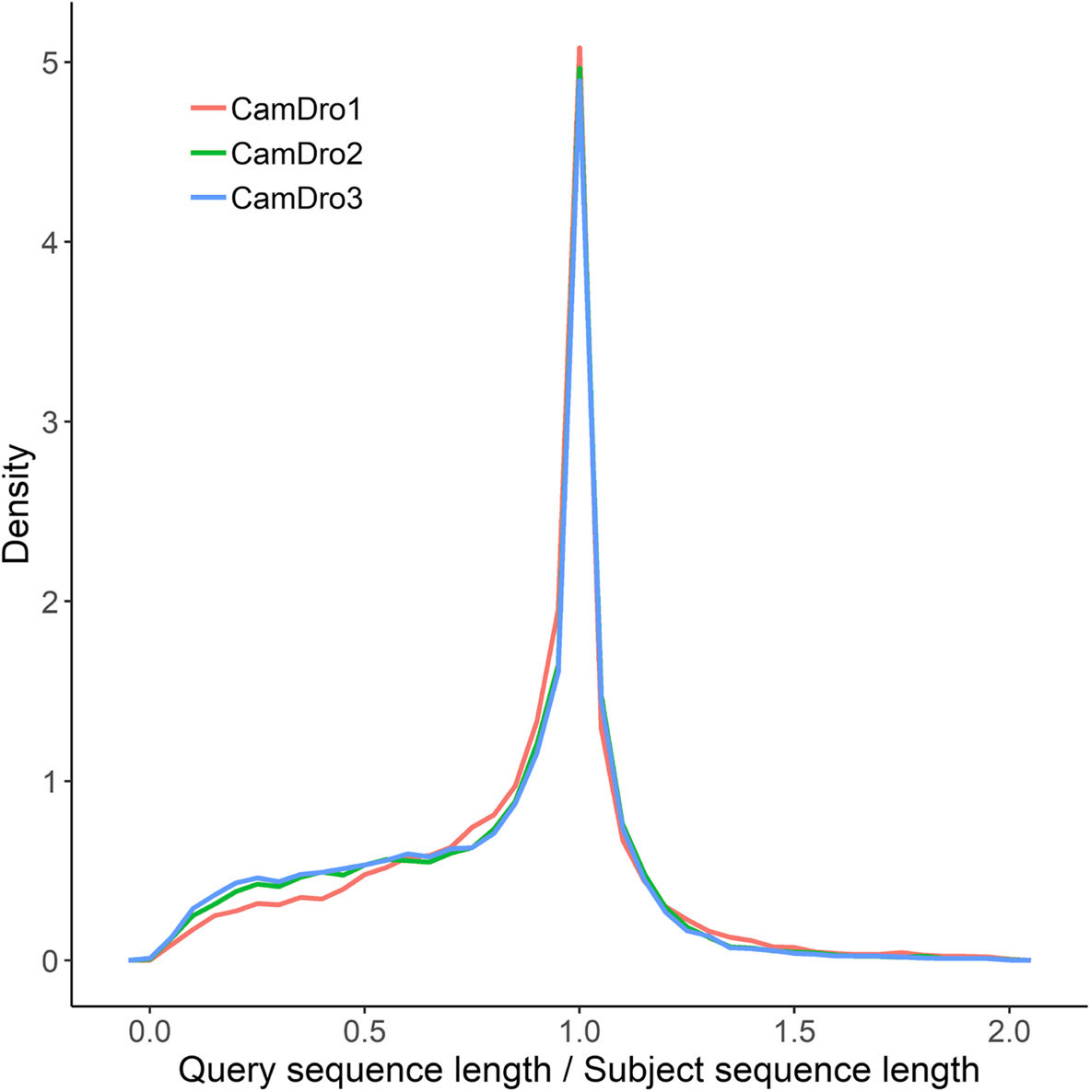
Frequency polygons of query sequence length (predicted proteins) divided by subject (UniProt/TrEMBL) sequence length for DIAMOND [13] mapped MAKER [14] predicted proteins against UniProt/TrEMBL release 2018_07 database for: (red line) the original North African dromedary genome (CamDro1), ([8]; GenBank accession: GCA_000803125.1); (green line) the North African dromedary genome after adding ~11x PacBio sequencing reads (CamDro2); and (blue line) CamDro3

**Fig. 2:**
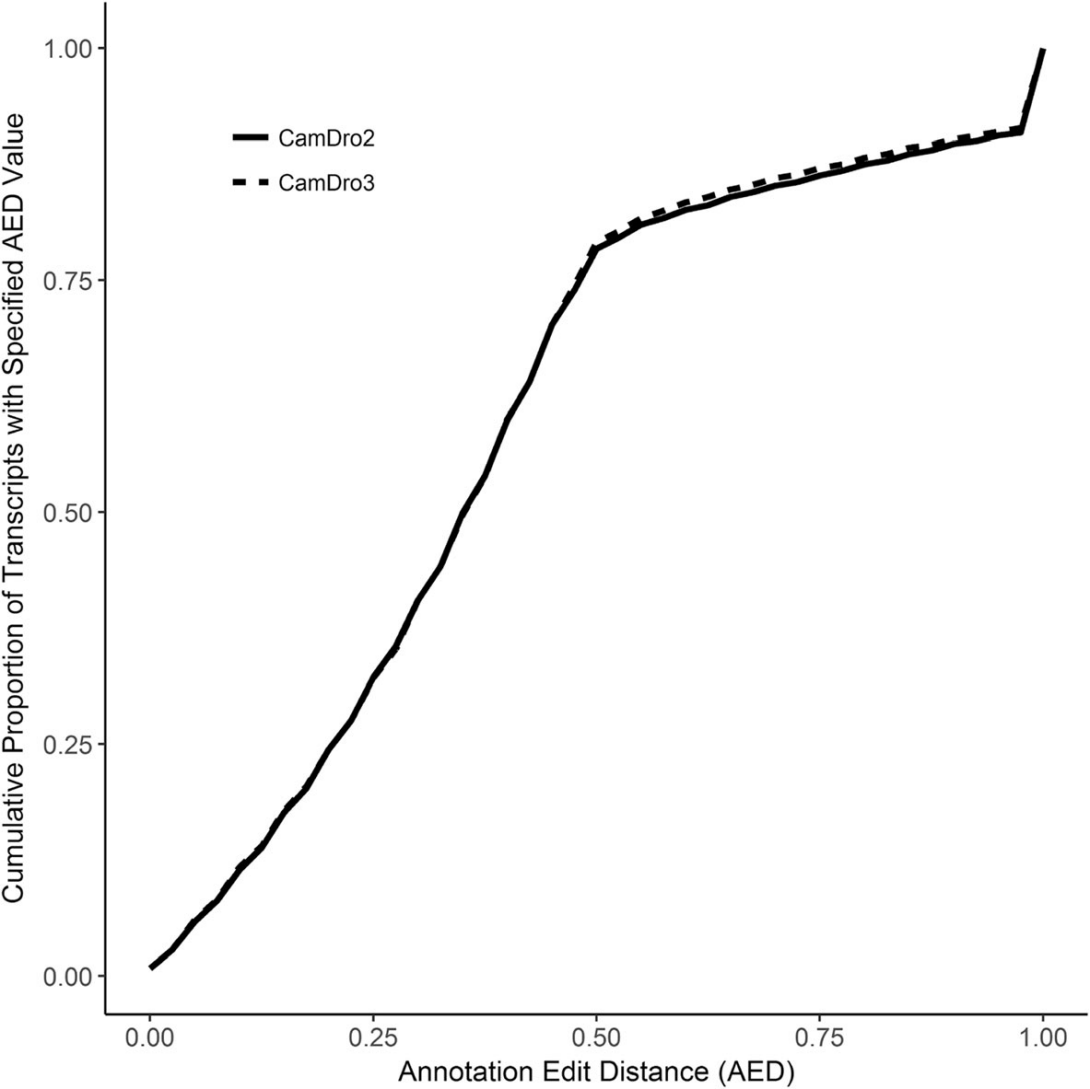
Cumulative proportion of transcripts with specific or lower annotation edit distance (AED) for CamDro2 (solid line) and CamDro3 (dashed line). CamDro2 had AED ≤ 0.50 for 78.4% transcripts, whilst MAKER run 2 had 79.1% transcripts with AED ≤ 0.50. Note that having a larger proportion of lower AED values indicates a genome annotation that is more congruent with the evidence used during the annotation process

In the CamDro3 annotation, we predicted 22,917 genes that produced 34,135 proteins, and 7.4% (1705) of genes had no assigned annotation. These numbers are slightly higher than for the CamDro2 assembly for which we had predicted 22,534 genes that produced 34,024 proteins, and 7.7% (1730) of genes had no assigned annotation [11]. We assessed if predicted proteins were truncated due to uncorrected indels introduced by PacBio reads by comparing the predicted protein length hit distribution of the CamDro1 assembly (Illumina only data, Fig. 1, red line), which should lack such PacBio specific error, to that of the CamDro2 (Fig. 1, green line) and CamDro3 assemblies (Fig. 1, blue line). First, predicted proteins from the CamDro1 assembly had 21,257 protein hits against the UniProt/TrEMBL database, and 11,671 (55%) hits were between 0.85 and 1.15 (query sequence length/ subject sequence length; Fig. 1). Second, predicted proteins from the CamDro2 assembly had 32,297 protein hits, and 17,341 (54%) were between 0.85 and 1.15 (Fig. 1). Third, predicted proteins for CamDro3 assembly had 32,427 protein hits, and 17,006 (52%) were between 0.85 and 1.15 (Fig. 1). This suggests that CamDro3 is similar to CamDro2 with respect to proportion of uncorrected PacBio indels, but the proportions of uncorrected indels are very low when compared to CamDro1. AEDs were slightly higher in CamDro3 versus CamDro2 (Fig. 2). For example, CamDro2 had AED values ≤0.5 for 78.4% transcripts versus 79.1% transcripts for CamDro3. Lower AED values indicate a better fit to the provided evidence when annotating a genome [15].

We predicted 22,223 genes that produced 33,153 proteins in CamDro3 using a more up to date set of proteins during annotation. These values were lower than when annotating CamDro3 using the same cDNA transcripts and proteins used for annotating CamDro2 possibly because there were fewer false genes predicted in the up-to-date annotation of CamDro3. Further, 8.46% (1879) genes produced proteins did not match UniProt/Swiss-Prot proteins. This value was higher than before, but we used UniProt/Swiss-Prot instead of the more comprehensive UniProt-TrEMBL protein database. The CamDro3 assembly and these annotations have been submitted to GenBank (GCA_000803125.3) and Dryad - see Data Accessibility Statement.

### Improved *Camelus bactrianus* and *Camelus ferus* genomes via reference-guided assembly

CamBac2 increased in size by 46,927,041 bases and had 1862 fewer scaffolds than CamBac1, and CamBac2’s N50 was nearly 8 times larger (Table 1). The longest contig in CamBac2 was more than 7 times larger than before. We have also predicted 19,491 genes that produced 25,95 proteins in CamBac2. Of these genes, 4.03% (786) did not match proteins from UniProt/Swiss-Prot. *Camelus bactrianus* had the second lowest mapping rates, after *B. taurus*. The CamBac2 assembly and these annotations have been submitted to Dryad - see Data Accessibility Statement.

**Table 1:** Assembly statistics for the CamBac1 (GCF_000767855.1) and CamFer1 (GCF_000311805.1) and after improvement (CamBac2 and CamFer2, respectively) with reference-guided assembly with Ragout [16] using Progressive Cactus [17] alignments to CamDro3 then filling in gaps with GapFiller [18]

Assembly				
	CamBac1	CamBac2	CamFer1	CamFer2
Total size	1,992,663,268	2,039,590,309	2,009,194,609	2,086,258,888
Gap length	13,666,687	57,965,943	23,778,176	99,159,843
**Scaffolds**				
Number	35,455	33,593	13,334	9158
Longest	46,538,883	122,729,119	15,735,958	123,639,755
N90^a^	1,821,536	24,994,512	341,469	25,431,863
L90^b^	255	29	1167	30
N50^a^	8,812,066	68,446,253	2,005,940	69,671,486
L50^b^	68	11	274	11
**Contigs**^**c**^				
Number	67,435	56,044	68,872	66,352
Longest	1,143,031	2,938,098	853,441	1,096,594
N90	29,656	43,365	16,267	16,886
L90	15,603	10,214	25,475	23,951
N50	139,019	219,031	90,263	97,198
L50	3963	2415	5814	5272
Single-copy BUSCOs^d^	3827	3835	3796	3816
Duplicated BUSCOs	22	18	48	32
Fragmented BUSCOs	164	157	175	168
Missing BUSCOs	91	94	85	88

^a^N90/N50 are the scaffold or contig lengths such that the sum of the lengths of all scaffolds or contigs of this size or larger is equal to 90/50% of the total assembly length

^b^L90/L50 are the smallest number of scaffolds or contigs that make up at least 90/50% of the total assembly length

^c^Using minimum gap length of 10 bp

^d^BUSCOs: Benchmarking Universal Single-Copy Orthologs [19] are mammalian BUSCOs from OrthoDB v. 9.1 genes [20]

CamFer2 was 77,064,279 bases larger and was organized into 4176 fewer scaffolds than CamFer1. CamFer2 had an N50 that was nearly 35 times larger than CamFer1’s N50 (Table 1). CamFer2’s longest contig was more than 2 times larger than CamFer1’s largest contig.

We predicted 19,192 genes that produced 19,192 proteins in CamFer2. Of these genes, 3.69% (708) did not match proteins from UniProt/Swiss-Prot. There were many structural variations (inversions and repeats) when comparing the assembled chromosomes of CamFer2 and the *C. ferus* genome assembly from Ming et al., [12] (Supplemental Fig. 2). Ultimately, these latter genomes have similar scaffold N/L50 values, but CamFer2 has much smaller contig N/L50 values because of more abundant and larger gaps in assembled chromosomes (Supplemental Table 1). The CamFer2 assembly and these annotations have been submitted to Dryad - see Data Accessibility Statement.

### Intra-specific genome-wide diversity

Mean coverage throughout the genomes of the three Old World camel species was not different among species (*F*_*2,22*_ = 0.1871*, P* = 0.8307; Table 2). The mean total number of SNPs was different among species (*F*_*2,22*_ = 64.943*, P* < 0.0001) as was the number of synonymous (*F*_*2,22*_ = 66.99*, P* < 0.0001) and non-synonymous SNPs (*F*_*2,22*_ = 113.25*, P* < 0.0001; Table 2). Mean total, synonymous, and non-synonymous SNPs were highest in Bactrian camels, followed by wild camels, then dromedaries. The mean number of insertions was different among species (*F*_*2,22*_ = 31.269*, P* < 0.0001) as was the mean number of deletions (*F*_*2,22*_ = 16.407*, P* < 0.0001; Table 2). Bactrian camels had a higher mean number of insertions than dromedaries and wild camels, which showed similar numbers of insertions. Bactrian camels had higher mean number of deletions, followed by wild camels, then dromedaries.

**Table 2:** Mean coverage and number of different types of variants per sample. DC for domestic Bactrian camel (*Camelus bactrianus*), Drom for dromedary (*Camelus dromedarius*), and WC for wild camel (*Camelus ferus*). SD for standard deviation

Sample	Mean Coverage	Total_SNPs	Synonymous SNPs	Non-synonymous SNPs	Insertions	Deletions
DC158	41.42	3,713,662	16,761	18,352	258,367	237,987
DC269	14.25	3,238,412	14,206	15,473	230,164	205,242
DC399	13.80	3,199,637	14,370	16,112	226,223	199,701
DC400	14.54	3,213,008	14,130	15,608	226,945	200,953
DC402	14.84	3,130,745	13,756	15,296	218,205	193,720
DC408	15.11	3,328,223	14,592	16,693	234,064	209,759
DC423	14.46	3,738,504	17,182	17,866	250,856	227,449
Drom439	14.30	1,929,784	8528	9135	163,100	147,765
Drom795	11.78	1,907,261	8600	9679	186,969	158,190
Drom796	14.23	1,991,649	8476	9193	170,719	156,795
Drom797	13.76	1,992,724	8945	9576	178,917	160,938
Drom800	40.73	1,500,998	6844	7255	140,148	122,312
Drom802	14.59	2,006,825	9311	10,122	188,392	166,360
Drom806	9.52	1,854,989	7944	8692	164,993	149,508
Drom816	10.33	1,929,982	8476	9263	173,380	154,757
Drom820	9.66	1,881,945	7694	8162	167,680	152,220
WC214	14.43	2,517,749	9919	10,071	157,630	162,297
WC216	12.86	2,654,274	11,040	10,871	170,009	176,405
WC218	14.22	1,825,617	7396	8026	109,795	107,655
WC219	14.04	2,707,996	11,187	11,038	173,685	179,297
WC220	14.92	2,707,716	11,067	10,982	170,579	179,365
WC247	14.06	2,956,856	11,567	11,235	189,010	196,986
WC303	41.54	2,937,692	11,625	11,313	189,408	204,838
WC304	14.67	2,748,380	11,047	10,844	180,435	186,048
WC305	14.05	2,704,263	10,599	10,520	176,820	181,412
Drom mean	15.43	1,888,462	8313	9009	170,478	152,094
Drom SD	9.7	154,355	729	867	14,512	12,552
DC mean	18.35	3,366,027	15,000	16,486	234,975	210,687
DC SD	10.2	252,904	1376	1210	14,409	16,125
WC mean	17.20	2,640,060	10,605	10,544	168,597	174,923
WC SD	9.1	334,004	1307	1017	24,154	28,002

### Heterozygosity rates in exons and introns

We assessed the heterozygosity rates in coding (exons) and noncoding (introns) regions, across multiple individuals. Heterozygosity means for all three species and coding/noncoding regions were all significantly different at the 0.05 level of significance. The results show that exons have significantly lower mean heterozygosity compared to introns in all three species, and that the domestic camel had the highest heterozygosity, followed by the dromedary and lastly the wild camel (DC: exons = 0.00110; introns = 0.00316; Drom: exons = 0.000983; introns = 0.00217; WC: exons = 0.000941; introns = 0.00231). These results are in accordance with what was found in Fitak et al. (2020) [21], although in Jirimutu et al. (2012) [9] the domestic camel genome had lower heterozygosity in the exonic regions compared to wild camel genome (though in the latter study the authors based their estimates on single individuals).

### Nucleotide diversity among Old World camels in immune response and intra-genic regions

**Fig. 3:**
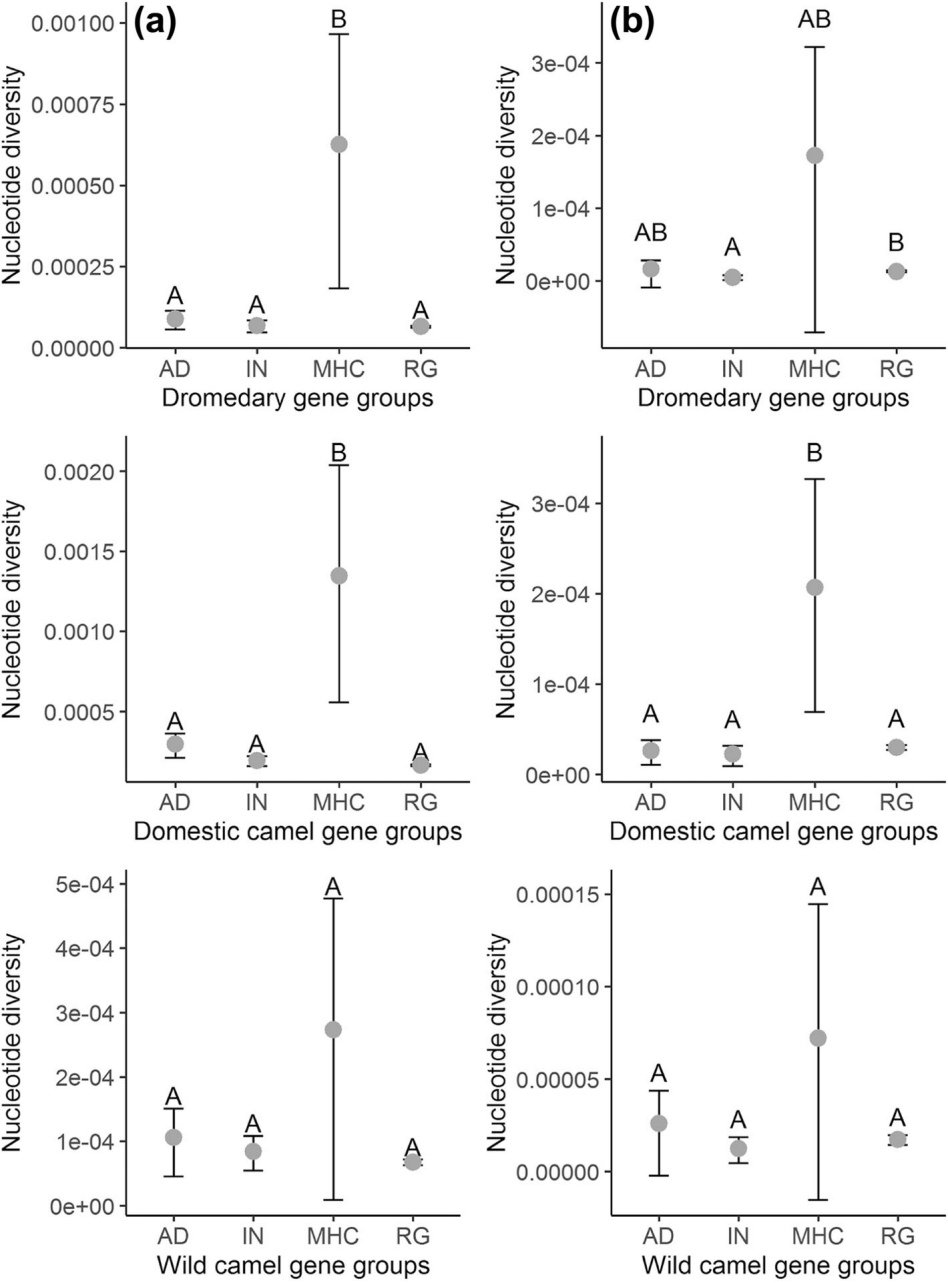
Means with 95% bootstrap confidence intervals (see Methods) of nucleotide diversity for alignments made with non-synonymous and synonymous SNPs and indels (**a**) and only non-synonymous SNPs (**b**) for: dromedary (*C. dromedarius*; top panel), domestic Bactrian camel (*C. bactrianus;* middle panel), and wild camel (*C. ferus*; bottom panel) gene groups. AD for adaptive genes, IN for innate genes, MHC for MHC class I and II genes, and RG for rest-of-genome genes. Rest-of-genome genes are those not classified as adaptive or innate genes (see Methods). Uppercase letters above upper 95% confidence limits indicate groups have different (non-matching letters) or not different (matching letters) means based on non-overlapping confidence intervals

After improving the three Old World camel genome assemblies, we assessed the nucleotide diversity in immune response and intra-genic (within gene) regions. When looking at non-synonymous and synonymous SNPs and indels altogether, mean nucleotide diversity was found not to differ significantly for adaptive, innate IR genes and the rest-of-genome genes, but to be higher in MHC class I and II genes in both dromedaries and domestic Bactrian camels (Fig. 3a; Table 3 for mean values and 95% bootstrap confidence limits). On the other hand, in wild camels, mean nucleotide diversity was not significantly different across gene types. When comparing nucleotide diversity per gene class in species pairs, mean MHC nucleotide diversity did not differ significantly for domestic Bactrian camels and dromedaries, as well as for wild camels and dromedaries, but differed between wild and domestic Bactrian camels, with the latter showing higher nucleotide mean diversity (Supplemental Fig. 3a; Table 3 for mean values and 95% bootstrap confidence limits). Innate and adaptive IR gene nucleotide diversity was statistically different between domestic Bactrian camels and the other two species, but the same between dromedaries and wild camels, while again Bactrian camels had a higher mean nucleotide diversity. Rest-of-genome gene nucleotide diversity was also higher for the Bactrian camel and different between this and the other two camel species.

**Table 3:** Means with 95% bootstrap confidence limits (CL, see Methods) of nucleotide diversity for alignments made with non-synonymous and synonymous SNPs and indels and only non-synonymous SNPs for: DROM (dromedary; *Camelus dromedarius*), DC (domestic Bactrian camel; *Camelus bactrianus*), and WC (wild camel; *Camelus ferus*) gene groups. AD for adaptive genes, IN for innate genes, MHC for MHC class I and II genes, and RG for rest of genome genes. Rest-of-genome-genes correspond to those genes which are not classified as adaptive or innate IR genes (see Methods)

Variant type	Species	Gene groups	Mean	95% lower CL	95% upper CL
SNPs and indels	DROM	MHC	6.26E-04	1.83E-04	9.65E-04
SNPs and indels	DROM	AD	8.81E-05	5.70E-05	1.14E-04
SNPs and indels	DROM	IN	6.81E-05	4.74E-05	8.49E-05
SNPs and indels	DROM	RG	6.55E-05	6.22E-05	6.87E-05
SNPs and indels	DC	MHC	1.35E-03	5.58E-04	2.04E-03
SNPs and indels	DC	AD	2.97E-04	2.11E-04	3.64E-04
SNPs and indels	DC	IN	1.94E-04	1.61E-04	2.23E-04
SNPs and indels	DC	RG	1.66E-04	1.60E-04	1.71E-04
SNPs and indels	WC	MHC	2.73E-04	9.06E-06	4.77E-04
SNPs and indels	WC	AD	1.06E-04	4.52E-05	1.51E-04
SNPs and indels	WC	IN	8.36E-05	5.45E-05	1.08E-04
SNPs and indels	WC	RG	6.71E-05	6.24E-05	7.13E-05
Non synonymous SNPs	DROM	MHC	1.72E-04	-7.09E-05	3.22E-04
Non synonymous SNPs	DROM	AD	1.58E-05	−8.83E-06	2.80E-05
Non synonymous SNPs	DROM	IN	4.79E-06	1.29E-06	7.42E-06
Non synonymous SNPs	DROM	RG	1.28E-05	1.13E-05	1.42E-05
Non synonymous SNPs	DC	MHC	2.07E-04	6.94E-05	3.27E-04
Non synonymous SNPs	DC	AD	2.63E-05	1.04E-05	3.80E-05
Non synonymous SNPs	DC	IN	2.26E-05	9.31E-06	3.17E-05
Non synonymous SNPs	DC	RG	2.97E-05	2.70E-05	3.25E-05
Non synonymous SNPs	WC	MHC	7.23E-05	−1.52E-05	1.45E-04
Non synonymous SNPs	WC	AD	2.61E-05	−2.17E-06	4.37E-05
Non synonymous SNPs	WC	IN	1.23E-05	4.52E-06	1.87E-05
Non synonymous SNPs	WC	RG	1.72E-05	1.45E-05	1.99E-05

On the other hand, when looking at only non-synonymous SNPs, dromedaries’ mean nucleotide diversity patterns were more difficult to interpret. Mean innate gene nucleotide diversity was lower than mean rest-of-genome gene nucleotide diversity, but mean innate gene nucleotide diversity was statistically not different from mean adaptive or MHC nucleotide diversity nor was mean rest-of-genome nucleotide diversity different from mean adaptive or MHC nucleotide diversity (Fig. 3b; Table 3 for mean values and 95% bootstrap confidence limits). In domestic Bactrian camels, mean nucleotide diversity was the same for adaptive, innate and the rest-of-genome genes, but different in MHC genes where it was the highest. On the other hand, in wild camels, all gene groups had statistically the same mean nucleotide diversity. For both MHC and adaptive IR genes, mean nucleotide diversity was the same among the three camel species (Supplemental Fig. 3b). For innate IR genes, Bactrian and wild camels had the same mean nucleotide diversities, whereas dromedaries had a different mean nucleotide diversity from the other camel species, but the same compared to wild camels. Finally, for the rest-of-genome genes group, all species had statistically different mean nucleotide diversities, where domestic Bactrian camels showed to have the highest values.

There were 46 identified single-domain heavy-chain immunoglobulin genes in the *Camelus ferus* assembly of Ming et al. [12]. Of those 46, annotations for 43 could be lifted over to CamDro3, 36 to CamBac2, and 39 to CamFer2, which mapped on chromosome 6 and on other scaffolds. Mean nucleotide diversity was not significantly different among dromedaries, domestic camels, or wild camels when using either alignments made with all SNPs and indels or only non-synonymous SNPs (see Supplemental Table 2 and Supplemental Fig. 4).

## Discussion

Despite its functional importance, the immunogenome of camels has received only limited attention, with work focusing on cytogenetic mapping in alpaca [22], the characteristics of single-domain heavy-chain antibodies [23] or specific mechanisms underlying the genetic diversity of T-cell receptors [24–26]. Dromedary and two-humped camels are important livestock species, well adapted to harsh conditions and resistant to devastating infections that threaten other livestock species in the same areas, like contagious pleuro-pneumonia [27] or foot-and-mouth disease in dromedaries [28]. Other infections have an important role in human health, such as the Middle East Respiratory Syndrome Coronavirus (MERS-CoV), for which dromedaries are potential reservoirs [29]. Variation in genetic diversity between innate and adaptive immunity genes is caused by differences in these gene groups’ mechanisms. While innate immunity is less specific and more executive, its genes are subject to purifying rather than to positive/balancing selection, whereas adaptive immunity is more focused on specific recognition of highly diverse antigens and its variability is exposed to different selective pressures [30, 31]. In this study, we compared the diversity in different groups of immune response genes with those found in intra-genic regions among the three Old World camel species, aiming to better understand to which selection pressures they might have been exposed. For this purpose, we first improved the three available Old World camelid genome assemblies.

### Old World Camelids genome assemblies´ improvement

We applied several computational techniques to improve previous assemblies. To begin with, we were able to greatly improve CamDro3 genome assembly from CamDro2. Compared with the previous version, the number of predicted gene proteins in the CamDro3 were lower, possibly because there were fewer false genes predicted. After correcting mis-assemblies by re-scaffolding CamDro2 and by using a different indel-polishing method, CamDro3 is now more complete, with fewer gaps and likely more accurate. Additionally, the reference-guided assembly process significantly improved the quality and contiguity of CamBac2 and CamFer2, as they are now more contiguous, and have fewer and longer scaffolds. By using a closely related genome to improve a draft assembly, it has a bigger impact on the final assembly, as well as the accuracy and completeness of a reference genome also contribute [32]. Although mean coverage throughout the genome was not different between species, mean total, synonymous, and non-synonymous SNPs, mean number of insertions and deletions were highest in domestic Bactrian camels compared to the other two species. These results might suggest that domestic Bactrian camels generally have higher genetic diversity than dromedaries and wild camels, as they might have experienced less severe demographic changes during domestication than dromedaries [33] and less recent population size reduction than the critically endangered wild camels [34].

### Nucleotide diversity in important immune gene groups

Old World camels are known to be resistant to serious infectious diseases that threaten other livestock species inhabiting the same geographical regions, although they may contract other poorly-studied diseases [35]. On the other hand, diseases of Camelidae are often difficult to deal with, having non-specific signs with a considerable economic impact [36]. Hence, as diversity in immune response gene regions may influence infectious disease susceptibility in populations, a better understanding of IR gene diversity will support camel breeding and sustainable management in countries of the Global South with large camel populations. As our data were not normally distributed and could not be transformed to approximate a normal distribution, we assessed differences in nucleotide diversity within species in different immune complexes of the genome by using a non-parametric bootstrapping method to estimate 95% confidence intervals of mean nucleotide diversity (Fig. 3 and Supplemental Fig. 3).

MHC class I and class II genes are amongst the most polymorphic genes studied in vertebrates [37]. Pathogen-mediated selection is widely held to be the major driving force in maintaining the high diversity at MHC loci [38]. In particular, the MHC diversity in populations is maintained by balancing selection [39]. According to the 95% confidence intervals derived from non-parametric bootstrap tests of mean nucleotide diversities, we observed that MHC (class I and II) genes had higher mean nucleotide diversity compared to all other gene groups, for two-humped camels, in both SNPs-indels and just non-synonymous SNPs analyses, and for dromedaries in SNP-indels analysis but not for only non-synonymous SNP analysis (Fig. 3). Previous research by Plasil et al., [4] showed that MHC nucleotide diversity within the three Old World species was generally low. In this case, the authors looked specifically into the antigen-binding sites and not to the complete genes where, according to our results, additional diversity appears to be present. The functional importance of this variation is currently unknown. However, it is important to acknowledge how particular pathogens affect immune genetic diversity and, vice versa, how genetic variation influences adaptation to emerging zoonosis, habitat fragmentation, and climate change [40]. MHC genes play an important role in the adaptive branch of the immune system and have been used extensively to estimate levels of adaptive genetic variation [41]. While innate immunity is an efficient first protection against many pathogens but rather less specific, adaptive (or acquired) immunity is a highly specific immune response, and its variability is subject to different selective pressures [30, 31]. Overall, mean nucleotide diversity was never different when comparing innate and adaptive IR gene groups in all three species, in both SNPs-indels and non-synonymous SNPs analyses.

When comparing nucleotide diversity among both two-humped camel species, wild camels had lower mean nucleotide diversity for both SNP and indels and non-synonymous SNP analyses, except for the MHC class I and II genes and for adaptive genes with non-synonymous SNPs (Supplemental Fig. 3). Moreover, in general, the domestic Bactrian camel had higher mean nucleotide diversity compared to the wild camel, except for the mean nucleotide diversity in adaptive genes with non-synonymous SNPs. One possible explanation for these results is that the wild camel suffered strong population declines leading to the current status of “critically endangered” species (by the International Union for Conservation of Nature (IUCN)). Thus, with the number of individuals decreasing, loss of genetic diversity is unfortunately real [42, 43]. Another possible explanation is that domestic Bactrian camels are under higher pathogenic pressure compared to the wild species. For example, Bactrian camels can be raised and herded with other domestic species (e.g., sheep or goat and sometimes cattle) and due to this fact, the animals are in contact with different pathogens that would not be present in the wild camels’ natural habitat [44]. This pathogenic pressure might have selected for higher diversity in domestic Bactrian camels, explaining the higher diversity in the immunogenome as well as in the rest of the genome. Nevertheless, we cannot discard the possibility that the demographic dynamics influenced the mean nucleotide diversity levels compared among species. Patterns of demographic changes across all three species demonstrated widespread population declines during the Pleistocene [21]. Principally in dromedaries, according to Lado et al. [45] and Fitak et al. [21], long-term population bottlenecks were detected, which probably reduced the nucleotide diversity even more in this species. Furthermore, there is the assumption that dromedaries have been domesticated from a relatively small population of wild one-humped camels, which already have been declining in numbers in a limited geographical area at the Southeast coast of the Arabian Peninsula [33]. However, the domestication of Bactrian camels might have occurred over a much larger geographic region, involving (genetically) more distant and diverse wild two-humped camels [12]. Our results suggest that the IR genes follow the same pattern of rest-of-the-genome genes where domestic Bactrian camels are more diverse throughout all classes of genes when compared to the endangered wild camel.

We also assessed the nucleotide diversity of single-domain heavy-chain immunoglobulin genes in our data. For that, we lifted the 46 heavy-chain immunoglobulin gene annotations from the Ming et al. 2020 [12] *Camelus ferus* genome assembly over to CamFer2, CamDro3, and CamBac2. However, we could not detect all 46 gene annotations on chromosome 6 and on other scaffolds as compared to *Camelus ferus* [12]. We were only able to recover 39 genes for CamFer2, 43 for CamDro3, and 36 for CamBac2. These lower numbers might be due to assembly quality as the contig (not scaffold) lengths are much longer in the Ming et al. [12] *Camelus ferus* assembly than in CamDro3, CamBac2, or CamFer2. Moreover, mean nucleotide diversity among dromedaries, domestic camels, and wild camels were not significantly different when using either alignments made with all SNPs and indels or only non-synonymous SNPs. In Ming et al. [12], the authors also compared the heavy-chain locus on chromosome 6 between the wild camel and alpaca (*Vicugna pacos*), and found that the gene content and order were very similar between the species. Interestingly, the alpaca, one of the four New World camel species, is evolutionarily the most closely related species to the Old World camels. Only recently, the most up-to-date chromosome-level reference genome assembly was released as VicPac3.1 [46]. Latest research shows that the genomic sequences of Natural Killer cell Receptor (NKR) genes were highly similar in both dromedary and domestic camel to alpaca sequences, as well as the organization of this genomic region [25]. Furthermore, high sequence similarity was observed for genes in the three different classes of MHC as well as MHC genes organization [46, 47].

## Conclusions

In this study, using different computational methods, we were able to improve genomic resources for *Camelus dromedarius*, *C. bactrianus* and *C. ferus*. Our data provides high-quality genome assemblies, which are now more contiguous and have fewer and longer scaffolds than the previous version, and are promising resources for the scientific community. Moreover, our results give new insights into the differences in mean nucleotide diversity in immune response genes within and among the three Old World camel species. From the three species, domestic Bactrian camels had the highest mean nucleotide diversity, and from the different functional gene groups, MHC genes had the highest mean diversity. Examining genetic variation in diverse immune genes in camels should be a priority, not only because camels are well adapted to extreme environments even in contact with different pathogens, but also because both domestic species are economically very important, and the wild two-humped camel is critically endangered. The data also showed that studies focused on functionally important parts of the genes, combined with analyses of selection at the molecular and population level, will be helpful to improve the understanding of the biology and evolution of these species. Altogether, this work not only opens doors for future immunogenome studies, but also serves as a reference to further genome assembly improvements using computational methods.

## Methods

### Previous dromedary genome assemblies

#### CamDro1

The original North African dromedary genome assembly (CamDro1) was created from a female dromedary “Waris” ([8]; GenBank accession: GCA_000803125.1). Briefly, two types of Illumina libraries were generated and sequenced: 500 bp (short-insert, 100 bp paired-end reads) and 5 Kbp (long-insert/mate-pair, 50 bp paired-end reads) libraries. Short- and long-insert reads were trimmed and, after short-insert reads error-correction, de novo assembled with ABYSS [48] with a k-mer value of 64.

#### CamDro2

Dovetail Genomics (Santa Cruz, California, USA) created and sequenced Chicago and Dovetail Hi-C libraries derived from the same dromedary “Waris” used in CamDro1. First, the CamDro1 assembly was scaffolded using Dovetail Chicago data run through the HiRise pipeline [49]. Next, the Chicago assembly was scaffolded with Hi-C data. Using a PacBio Sequel sequencer, 11x long-read coverage were generated ([11]; Sequence Read Archive (SRA) accession: SRP050586) and PBJelly [50] was used to fill in gaps in the Hi-C assembly. PBJelly assembly was polished with Pilon [51] employing the same trimmed and error-corrected Illumina short-insert sequences used for the de novo assembly of CamDro1 by Fitak et al. ([8]; SRA accession: SRR2002493). Gaps present in the Pilon assembly were then filled with ABYSS Sealer [52]. Finally, the ABYSS assembly was polished with Pilon once again. This assembly is referred to as CamDro2 ([11]; GCA_000803125.2).

### Improving the dromedary genome assembly: CamDro3

The CamDro2 assembly was re-scaffolded using the original Dovetail Chicago and Hi-C reads with the HiRise pipeline. We then filled in gaps using our PacBio long-reads ([11]; SRA accession: SRP050586), running PBJelly v. 15.8.24 twice. Instead of polishing the assembly with Pilon, we used a standard variant calling workflow, which increased RNA-Seq reads mapping rates relative to the Pilon-polished assembly (Table 4). Briefly, we first mapped trimmed and error-corrected Illumina short-insert sequences ([8]; Sequence Read Archive accession: SRR2002493) using BBMap v. 38.12 (https://sourceforge.net/projects/bbmap/) with the vslow and usejni settings to the PBJelly assembly. We then sorted and indexed the resulting BAM file with Sambamba v. 0.6.7 [55] and called variants with CallVariants v. 38.12 (https://sourceforge.net/projects/bbmap/). We finally used BCFtools v. 1.2 (http://samtools.github.io/bcftools/) to generate a consensus sequence for which we filled in gaps using ABySS Sealer v. 2.1.0 [52] using default settings except for a bloom filter size of 40 GB and multiple *K* values from 90 to 20 in increments of 10. We refer to this as the CamDro3 assembly (GCA_000803125.3).

**Table 4:** Assembly statistics for the CamDro2; CamDro3 (Pilon) using one round of Pilon [51] for polishing; and CamDro3 (BBMap) using one round of variant calling with BBMap (https://sourceforge.net/projects/bbmap/) for polishing. Note that CamDro3 (BBMap) was chosen over CamDro3 (Pilon) as the final version of CamDro3 because of better BUSCO and RNA-Seq mapping percentages

	Assembly		
	CamDro2	CamDro3 (Pilon)	CamDro3 (BBMap)
Total size	2,154,386,959	2,194,229,671	2,169,346,739
Gap length	20,603,579	17,930,821	17,043,352
**Scaffolds**			
Number	23,439	21,070	21,070
Longest	124,992,380	125,472,505	124,715,342
N90^a^	4,922,612	25,062,887	24,767,672
L90^b^	31	32	32
N50^a^	75,021,453	70,557,636	70,369,702
L50^b^	11	12	11
**Contigs**^**c**^			
Number	45,969	41,934	53,085
Longest	9,490,880	14,412,615	2,012,572
N90	177,587	202,272	49,444
L90	1944	1436	10,023
N50	1,333,162	1,961,815	236,380
L50	423	303	2637
Single-copy BUSCOs^d^	3851	3853	3852
Duplicated BUSCOs	24	23	25
Fragmented BUSCOs	133	132	134
Missing BUSCOs	96	96	93
RNA-Seq Mapping Percentage^e^	88.30	90.36	92.04

^a^N90/N50 are the scaffold or contig lengths such that the sum of the lengths of all scaffolds or contigs of this size or larger is equal to 90/50% of the total assembly length

^b^L90/L50 are the smallest number of scaffolds or contigs that make up at least 90/50% of the total assembly length

^c^Using minimum gap length of 25 bp

^d^BUSCOs: Benchmarking Universal Single-Copy Orthologs [19] are mammalian BUSCOs from OrthoDB v. 9.1 genes [20]

^e^Overall mapping rates using HiSat v. 2.1.0 [53] of dromedary RNA-Seq reads from Sequence Read Archive accession: SRP017619 and Alim et al. [54]

### RNA-Seq analysis of dromedary

To assess the quality of the new assembly, we aligned 10 sets of paired-end RNA-Seq reads (Alim et al., 2019) to the original assembly (CamDro1), to CamDro2, the new assembly (CamDro3), and to several controls: *C. dromedarius* (RefSeq version - GCA_000767585.1), *C. bactrianus* (GCA_000767855.1), *C. ferus* (GCA_000311805.2) and *Bos taurus* (cattle) (GCA_000003055.3). The 10 RNA-Seq datasets were part of a 2 × 2 factorial experiment: summer vs. winter seasons and supraoptic nucleus (SON) vs. neurointermediate lobe (NIL) brain tissues, with *n* = 3 replicates in each class. Tissue was homogenized and extracted usingTrizol/chloroform (ThermoFisher), and purified with the RNeasy MiniKit (Qiagen). The library template was prepared using a ribosome depletion protocol (Ribo-Zero Gold; Illumina) and libraries prepared using TruSeq Stranded protocol (Illumina). Samples were multiplexed into lane pools with an 8pM concentration and sequenced (100 bp paired-end reads with an average 134 bp insert size) to a depth of > 35 million reads using an Illumina HiSeq 2500. Two of the 12 replicates were rejected for insufficient quality. We used Tophat v. 2.0.9 [56] with default settings to align reads to each genome and report overall alignment rate (default output of Tophat) within each class. For chromosome mapping we then used blastn v. 2.2.31+ [57] to map 4981 probe sequences assigned to *Vicuna (Lama) pacos* chromosomes [11, 22] to CamDro3 assembly scaffolds. We followed the same procedure as Elbers et al., [11].

### Annotation to compare CamDro3 to CamDro2

To compare CamDro2 and CamDro3 assemblies, we annotated CamDro3 following the same steps used to annotate CamDro2 [11]. Briefly, we annotated scaffolds greater than 10 Kbp with MAKER v. 2.31.9 [14, 58]. We masked repetitive regions with RepeatMasker v. open-4.0.7 against the entire Dfam_Consensus release 20,170,127 database. We included ab initio gene predictions from GeneMark-ES 4.33 [59], expressed sequence tag (EST) transcripts, and protein sequences. For ESTs, we assembled transcripts from two dromedary transcriptome experiments (SRA accession: SRP017619 and [54]). We performed adapter and quality trimming on raw demultiplexed paired-end reads using BBDuk v. 37.25, using the following settings: ktrim = r, k = 23, mink = 11, hdist = 1, tpe, tbo, qtrim = rl, trimq = 15. We then mapped quality and adapter trimmed reads to the CamDro3 assembly using HiSat v. 2.1.0 [53] using a maximum intron length of 100,000 and the “dta” option. Reads were assembled into transcripts using StringTie v. 1.3.3b [60] and extracted using Gffread v. 0.9.9 (https://github.com/gpertea/gffread). For proteins, we combined predicted proteins from *B. taurus*, *C. bactrianus*, and *V. pacos* (GenBank accessions [NCBI annotation release]: GCF_000003055.6 [105], GCF_000311805.1 [100], and GCF_000164845.2 [101], respectively). We also included MAKER predicted proteins with an annotation edit distance (AED) < 0.75 from the CamDro1 assembly [8]. We trained Augustus v. 3.3 [61] using BUSCO v. 3.0.2 (Simão et al., 2015) searching for Eukaroyota OrthoDB v. 9.1 genes [20]. We used a *C. dromedarius* specific repeat library created with RepeatModeler v. open-1.0.10 (http://www.repeatmasker.org) with the CamDro3 as input. We filtered the repeat library from RepeatModeler to remove known UniProt/SwissProt v. 2017_10 [62] proteins using ProtExcluder v. 1.1 [63]. We only retained genes, transcripts, and proteins with AED ≤ 0.50. Next, we predicted putative gene function with DIAMOND v. 0.9.19 [13] searches against the UniProt/TrEMBL release 2018_07 database using an e-value cutoff of 1e^−^ 6. For the CamDro1, CamDro2, and CamDro3 assemblies, we also mapped proteins predicted by MAKER against the same UniProt/TrEMBL database using DIAMOND and generated a frequency polygon of the query sequence length (predicted proteins) divided by the subject sequence length (UniProt/TrEMBL proteins) to assess if predicted proteins were truncated (query sequence length divided by the subject sequence length < 1.0) due to uncorrected insertions/deletions (indels) introduced by PacBio reads that might interrupt reading frames affecting protein translation [64].

### Reference-guided assembly of the domestic Bactrian and wild camel genomes

We used CamDro3 in a reference-guided assembly strategy implemented with Ragout v. 2.0 [16] to upgrade the *C. bactrianus* (CamBac1, GCF_000767855.1, [10]) and *C. ferus* (CamFer1, GCF_000311805.1, [9]) genome assemblies to chromosome-level scale. Briefly, we used default settings in Progressive Cactus v. Github commit c4bed56c0cd48d23411038acb9c19bcae054837e [17, 65] to generate HAL (hierarchical alignment format) alignments between CamDro3 and CamBac1 or CamDro3 and CamFer1, and then used Ragout with the “refine” and “small synteny block” settings to convert the alignments to FASTA, upgrading the CamBac1 and CamFer1 assemblies to CamBac2 and CamFer2, respectively. Before alignment with Progressive Cactus, we repeat-masked CamDro3 with RepeatMasker v. open-4.0.8 (http://www.repeatmasker.org) against the mammal repeats from RepBase RepeatMaskerEdition-20,181,026 [66]. We filled in gaps in CamBac2 and CamFer2 with GapFiller v. 1.10 [18] using default settings and BowTie [67] as the aligner. The paired-end reads used to fill in gaps were the original Illumina short-reads used in assembly with an insert size less than or equal to 800 bases (For CamBac2 SRA accessions: SRR1552325, SRR1552327, SRR1552330, SRR1552336, SRR1552341, SRR1552346, SRR1552347, and SRR1552348;for CamFer2 SRA accession: SRR671683), which we trimmed with BBDuk v. 37.76 (https://sourceforge.net/projects/bbmap/), using the following settings: ktrim = r, k = 23, mink = 11, hdist = 1, tpe, tbo, qtrim = rl, trimq = 15, ref. = bbmap-37.76/resources/adapters.fa. We used assemblathon_stats.pl (http://korflab.ucdavis.edu/Datasets/Assemblathon/Assemblathon2/Basic_metrics/assemblathon_stats.pl) to compare assembly statistics between CamFer2 and the *C. ferus* genome assembly from Ming et al. [12] using a genome size of 2.1 Gbp. To assess the level of disagreement between CamFer2 and *C. ferus* genome assembly from Ming et al. [12], we made a whole genome alignment with Minimap2 v. 2.17 [68] using the “asm5” preset. We then used D-GENIES [69] to generate a dot plot for the alignment by using the contig sorting function and filtering alignments for strong precision. Chromosomal synteny between the wild camel and dromedary was analyzed by Ming et al. [12] after whole-genome alignment between *C. ferus* genome assembly (new-CamFer) and CamDro3, where assignment of the chromosome nomenclature between these species was similar, with only few structural differences at the megabase (Mbp) scale. Synteny is likely highly conserved between wild camel and dromedary, and domestic Bactrian and dromedary.

### Most up to date annotation for CamBac2, CamFer2, CamDro3

To get the most up to date annotation for CamBac2, CamFer2, and CamDro3, we annotated scaffolds greater than 10 Kbp in these assemblies with MAKER v. 2.31.10. We masked repetitive regions with RepeatMasker v. open-4.0.7 against the entire Dfam_Consensus release 20,170,127 database. We included ab initio gene predictions from GeneMark-ES v. 4.38, EST transcripts, and protein sequences. For CamDro3 ESTs but CamBac2 and CamFer2 alternative ESTs, we assembled transcripts from two dromedary transcriptome experiments (SRA accession: SRP017619 and [54]). We performed adapter and quality trimming on raw demultiplexed paired-end reads using BBDuk v. 37.25, using the following settings: ktrim = r, k = 23, mink = 11, hdist = 1, tpe, tbo, qtrim = rl, trimq = 15. We then mapped quality and adapter trimmed reads to the CamDro3 assembly using HiSat v. 2.1.0 using a maximum intron length of 100,000 and the “dta” option. Reads were assembled into transcripts using StringTie v. 1.3.3b and extracted using Gffread v. 0.9.9. For CamBac2 ESTs but CamDro3 and CamFer2 alternative ESTs, we processed transcriptome reads from *C. bactrianus* (SRA accessions: SRP014573 and SRP148535) with HiSat, StringTie, and Gffread as before but mapped quality controlled reads to the CamBac2 assembly. For proteins, we combined predicted proteins from *B. taurus*, *C. bactrianus*, *C. dromedarius, C. ferus*, and *V. pacos* (GenBank accessions (NCBI annotation release): GCF_002263795.1 (106), GCF_000767585.1 (100), GCF_000767855.1 (100), GCF_000311805.1 (101), and GCF_000164845.2 (101), respectively). We trained Augustus v. 3.3.2 using BUSCO v. 3.0.2 searching for Eukaroyota OrthoDB v. 9.1 genes in CamDro3, CamBac2, and CamFer2. We used a *C. dromedarius, C. bactrianus,* or *C. ferus* specific repeat library created with RepeatModeler open-1.0.10 with the CamDro3, CamBac2, or CamFer2 assemblies as input, respectively. We filtered each repeat library from RepeatModeler to remove known UniProt/Swiss-Prot release 2018_11 proteins using ProtExcluder v. 1.1. We only retained genes, transcripts, and proteins with AED ≤ 0.50. Next, we predicted putative gene function with blastp v. 2.2.31+ [57] searches against the UniProt/Swiss-Prot release 2018_11 database using an e-value cutoff of 1e^−^ 6.

### Variant calling

From whole-genome sequencing reads (100-bp Illumina paired end reads) of 25 Old World camels [21], we removed adapter sequences and reads with > 10% uncalled bases and/or > 50% of bases with a Phred-scaled quality score < 4. We also trimmed reads with PoPoolation v. 1.2.2 [70], where low-quality bases with a Phred score below 20 at the ends of the reads were removed. We converted base quality scores from Phred 64 to Phred 33 encoding and performed quality trimming with Repair v. 38.39 (https://sourceforge.net/projects/bbmap/) using the qtrim = rl and trimq = 15 settings. We mapped quality and adapter trimmed paired-end reads for *C. bactrianus*, *C. dromedarius*, and *C. ferus* individuals to the CamBac2, CamDro3, and CamFer2 references, respectively with BWA-MEM v. 0.7.17 [71, 72]. We converted SAM files to BAM files with SAMtools v. 1.9 [73], then cleaned, sorted, added read groups, and marked duplicates with Picard v. 2.18.10 (http://broadinstitute.github.io/picard). We called variants for each species separately with CallVariants v. 38.39 (https://sourceforge.net/projects/bbmap/), keeping only SNPs and indels with quality scores greater than or equal to 27. We predicted what SNP alleles might be synonymous or non-synonymous using snpEff v 4.0e [74].

We calculated coverage metrics with mosdepth v. 0.2.6 [75] with the settings “-n --fast-mode and --by 500”. We used R v. 3.6.0 to test for differences in mean coverage, total number of SNPs, number of synonymous SNPs, number of non-synonymous SNPs, number of insertions, and number of deletions within species with the “lm” and “anova” base functions. For all models, we used a Benajimini-Hochberg post-hoc test [76] implemented in glht and summary functions in the R package multcomp v. 1.4–10 [77].

### Heterozygosity rates in exons and introns

We predicted intron regions for gene annotations of CamDro3, CamBac2, and CamFer2 using Genome Tools v. 1.5.8 [78] with the gff3 function and -addintrons -retainids options. We then generated gene annotation files of only exons or introns for each camel species. We filtered the VCF files for each individual to retain only heterozygous SNPs. We then used BEDTools intersect v. 2.29.0 [79] to count the number of heterozygous SNPs for each individual (*n* = 25) in the exons or introns across the genome. We estimated heterozygosity as the number of heterozygous SNPs in the exons or introns of a given gene for a given individual divided by gene length.

We used the lm function in R 3.6.3 using heterozygosity as the dependent variable and the interaction of species and whether heterozygosity was estimated from exons or introns (hereafter exons or introns) as the independent variable. Residuals needed to be log10 transformed to be normally distributed. We used a generalized least squares variation of ANOVA (hereafter ANOVA [80]) as our transformed data did not have homogeneous variance. To control for heterogeneous variance, we used weights as “varIdent=(1|interaction of species, and exons or introns)” implemented with the gls function in the R package nlme v. 3.1–147 [81]. We used a Benajimini-Hochberg post-hoc test as before implemented with the glht and summary functions in the R package multcomp v. 1.4–13 and the cld function in multcomp with the options level = 0.05 and decreasing = T to determine if means for all species for exons and introns were significantly different at the 0.05 level.

### Nucleotide diversity

Two comparisons of nucleotide diversity were made, (i) between functionally different gene groups within each species: innate immune response genes, adaptive immune response genes, MHC class I and II genes, and rest-of-genome genes, and (ii) between Old World camel species: domesticated dromedaries and Bactrian camels, and wild camels among gene groups.

To test for differences in genetic variation among functionally different gene groups, we performed blastp searches of CamBac2, CamFer2, and CamDro3 predicted proteins against UniProt/Swiss-Prot release_2018_11 proteins to assign gene ontology terms, and filtered these gene/GO term lists by the gene ontology terms “innate immune response” and “adaptive immune response” using the rGO2TR package [82]. For MHC class I and class II genes, we filtered the GFF3 (General Feature Format) files of gene annotations manually. For the rest-of-genome gene group, we examined genes that were not assigned to either the innate or adaptive immune response gene groups. We used BCFtools v. 1.9 to generate a consensus sequence with IUPAC codes for each individual against its respective reference genome for each gene being analyzed and made a multiple sequence alignment for each gene and species with FSA v. 1.15.9 [83] with MuMmer v. 4.0.0beta2 [84] for long alignments. Finally, we calculated nucleotide diversity for entire gene sequence multiple sequence alignments (each species separately) using the R package Pegas’s “nuc.div” function [85]. We used R v. 3.6.3 to test for differences in mean nucleotide diversity within species among gene groups. For this we compared the 95% confidence intervals of the mean estimated with the boot.ci function’s “basic” confidence interval method based on 1000 “ordinary” simulations (i.e., non-parametric bootstraps) implemented with the boot function from the R package boot v. 1.3–24 [86]. We chose to use non-parametric inference as the residuals could not be transformed to approximate a normal distribution, precluding the use of traditional ANOVA/linear model testing for differences in means.

For analyzing differences in mean nucleotide diversities within gene groups but among species, we used the same procedures as before but with the explanatory variable “species” (dromedary, domestic Bactrian camel, or wild camel) and response variable “nucleotide diversity” (adaptive, innate, MHC, or rest-of-genome genes). In addition to nucleotide diversity, estimated with gene consensus sequences made with non-synonymous and synonymous SNPs and indels, we also repeated all steps above using only non-synonymous SNPs (indels and synonymous SNPs were not included).

Interestingly, camelids (New World and Old World camels) produce homodimeric heavy-chain immunoglobulins (hcIGs [87];) without a light chain and with the antigen-binding fragment reduced to a single heavy-chain variable domain (VHH), in addition to the conventional antibodies [88]. To assess the nucleotide diversity of single-domain heavy-chain IG genes in our data, we first downloaded the scaffold.fasta.gz (Ming et al.’s [12] *Camelus ferus* genome assembly) and IGH.gff (heavy-chain immunoglobulin gene annotations) from https://figshare.com/articles/Data_from_Chromosome-level_assembly_of_wild_Bactrian_camel_genome_reveals_organization_of_immune_gene_loci/11297489. We then lifted over the *Camelus ferus* IGH.gff gene annotations assembly [12] to CamDro3, CamBac2, and CamFer2 using Liftoff Github commit #77b7c4c91b294737d18d7a76e3611d279bebea6e [89]. We repeated previous nucleotide diversity assessment steps as described above (see *Nucleotide diversity*) using the new lifted over annotations. As we could not transform data to have residuals with a normal distribution, we followed analysis steps as before, except that we used R v. 3.6.3 along with the R package boot v. 1.3–25 [86], and compared mean nucleotide diversity in heavy-chain immunoglobulin genes among dromedaries, domestic camels, and wild camels.

## Supplementary information

**Additional file 1: Supplemental Table 1.** Assembly statistics for the CamFer2 and the *Camelus ferus* genome (new-CamFer) assembly from Ming et al. (2020b) using a genome size of 2.1 Gbp.

**Additional file 2: Supplemental Table 2.** Means with 95% bootstrap confidence limits (CL, see Methods) of nucleotide diversity for alignments made with non-synonymous and synonymous SNPs and indels and only non-synonymous SNPs in HC (heavy-chain) immunoglobulin genes in DC (domestic camel), DROM (dromedary), and WC (wild camel).

**Additional file 3: Supplemental Figure 1.** RNA-Seq mapping rates.

**Additional file 4: Supplemental Figure 2**. D-GENIES (Cabanettes & Klopp, 2018) dot plot made with Minimap2 [68] whole-genome alignment between CamFer2 and the *Camelus ferus* genome (new-CamFer) assembly from Ming et al., [12]. Contigs are sorted and matches are filtered using the strong precision setting in D-GENIES.

**Additional file 5: Supplemental Figure 3.** Means with 95% bootstrap confidence intervals (see Methods) of nucleotide diversity for alignments made with non-synonymous and synonymous SNPs and indels (a) and only non-synonymous SNPs (b): MHC class I and II genes (top panel), innate (second panel), adaptive (third panel), and the rest of genome genes (bottom panel) for: DROM (dromedary, *C. dromedarius*), DC (domestic Bactrian camel, *C. bactrianus*), and WC (wild camel, *C. ferus*). Uppercase letters above upper 95% confidence limits indicate groups have different (non-matching letters) or not different (matching letters) means based on non-overlapping confidence intervals.

**Additional file 6: Supplemental Figure 4.** Means with 95% bootstrap confidence intervals (see Methods) of nucleotide diversity for alignments made with (left) non-synonymous SNPs, (right) all SNPs and indels in HC (heavy-chain) antibody (immunoglobulin) genes in DC (domestic camel), DROM (dromedary), and WC (wild camel). Uppercase letters above upper 95% confidence limits indicate groups have different (non-matching letters) or not different (matching letters) means based on non-overlapping confidence intervals.

## Data Availability

CamDro3 is available from NCBI GenBank (GCA_000803125.3) and NCBI RefSeq (GCF_000803125.2). Our CamDro3/CamBac2/CamFer2 gene annotations, predicted mRNA and proteins, and assemblies for gene annotations are available from Dryad (10.5061/dryad.qv9s4mwb3). Raw VCF files (snp and indel variants) for each camel are also available in the Dryad repository. Example scripts and code for analyses are available from the Dryad repository.

## Abbreviations

AED: Annotation edit distance
ANOVA: Analysis of Variance
DC: Domestic Bactrian camel (*Camelus bactrianus*)
Drom: Dromedary (*Camelus dromedarius*)
EST: Expressed sequence tag
hcIGs: Homodimeric heavy-chain immunoglobulins
Indels: Insertions/deletions
IR: Immune-response
IUPAC: International Union of Pure and Applied Chemistry
Kbp: Kilo base pairs
Mbp: Mega base pairs
MHC: Major histocompatibility complex
NIL: Neurointermediate lobe
NKR: Natural Killer cell Receptor
RNA-Seq: Ribonucleic acid sequencing
SD: Standard deviation
SNP: Single nucleotide polymorphism
SON: Supraoptic nucleus
SRA: Sequence Read Archive
VHH: Single heavy-chain variable domain
WC: Wild camel (*Camelus ferus*)
