# Supplementary material for: Nucleotide diversity of functionally different groups of immune response genes in Old World camels based on newly annotated and reference-guided assemblies
Source: BMC Genomics. 2020 Sep 3;21:606. doi: 10.1186/s12864-020-06990-4 (PMC7468183; doi:10.1186/s12864-020-06990-4)
Supplement: Supplementary file 1 — Additional file 1: Supplemental Table 1. Assembly statistics for the CamFer2 and the Camelus ferus genome (new-CamFer) assembly from Ming et al. (2020b) using a genome size of 2.1 Gbp. [file 12864_2020_6990_MOESM1_ESM.docx]

**Supplemental Table 1.** Assembly statistics for the CamFer2 and the *Camelus ferus* genome (new-CamFer) assembly from Ming et al. (2020b) using a genome size of 2.1 Gbp.

| **Assembly** | **new-CamFer** | **CamFer2** |
| --- | --- | --- |
| Number of scaffolds | 2057 | 9158 |
| Total size of scaffolds | 2087077831 | 2086258888 |
| Total scaffold length as percentage of assumed genome size | 99.4 | 99.3 |
| Longest scaffold | 122453268 | 123639755 |
| Shortest scaffold | 5 | 200 |
| Number of scaffolds > 1K nt | 1997 | 8465 |
| Percentage of scaffolds > 1K nt | 97.1 | 92.4 |
| Number of scaffolds > 10K nt | 1175 | 619 |
| Percentage of scaffolds > 10K nt | 57.1 | 6.8 |
| Number of scaffolds > 100K nt | 99 | 61 |
| Percentage of scaffolds > 100K nt | 4.8 | 0.7 |
| Number of scaffolds > 1M nt | 43 | 42 |
| Percentage of scaffolds > 1M nt | 2.1 | 0.5 |
| Number of scaffolds > 10M nt | 36 | 36 |
| Percentage of scaffolds > 10M nt | 1.8 | 0.4 |
| Mean scaffold size | 1014622 | 227807 |
| Median scaffold size | 11758 | 1634 |
| N50 scaffold length | 76025729 | 69671486 |
| L50 scaffold count | 11 | 11 |
| NG50 scaffold length | 76025729 | 69671486 |
| LG50 scaffold count | 11 | 11 |
| N50 scaffold - NG50 scaffold length difference | 0 | 0 |
| scaffold %A | 29.17 | 27.96 |
| scaffold %C | 20.83 | 19.65 |
| scaffold %G | 20.82 | 19.66 |
| scaffold %T | 29.17 | 27.98 |
| scaffold %N | 0.01 | 4.75 |
| scaffold %non-ACGTN | 0 | 0 |
| Number of scaffold non-ACGTN nt | 0 | 0 |
| Percentage of assembly in scaffolded contigs | 97.6 | 99 |
| Percentage of assembly in unscaffolded contigs | 2.4 | 1 |
| Average number of contigs per scaffold | 2.1 | 6.7 |
| Average length of break (>25 Ns) between contigs in scaffold | 100 | 1884.878498 |
| Number of contigs | 4402 | 61715 |
| Number of contigs in scaffolds | 2382 | 53692 |
| Number of contigs not in scaffolds | 2020 | 8023 |
| Total size of contigs | 2086843331 | 1987191559 |
| Longest contig | 26533942 | 1096594 |
| Shortest contig | 5 | 4 |
| Number of contigs > 1K nt | 4342 | 54533 |
| Percentage of contigs > 1K nt | 98.6 | 88.4 |
| Number of contigs > 10K nt | 3469 | 29287 |
| Percentage of contigs > 10K nt | 78.8 | 47.5 |
| Number of contigs > 100K nt | 1123 | 5170 |
| Percentage of contigs > 100K nt | 25.5 | 8.4 |
| Number of contigs > 1M nt | 389 | 2 |
| Percentage of contigs > 1M nt | 8.8 | 0 |
| Number of contigs > 10M nt | 32 | 0 |
| Percentage of contigs > 10M nt | 0.7 | 0 |
| Mean contig size | 474067 | 32199 |
| Median contig size | 28692 | 8750 |
| N50 contig length | 5365398 | 104662 |
| L50 contig count | 112 | 4862 |
| NG50 contig length | 5355949 | 97024 |
| LG50 contig count | 113 | 5422 |
| N50 contig - NG50 contig length difference | 9449 | 7638 |
| contig %A | 29.17 | 29.36 |
| contig %C | 20.83 | 20.63 |
| contig %G | 20.82 | 20.64 |
| contig %T | 29.17 | 29.37 |
| contig %N | 0 | 0 |
| contig %non-ACGTN | 0 | 0 |
| Number of contig non-ACGTN nt | 0 | 0 |
