# Supplementary material for: Nucleotide diversity of functionally different groups of immune response genes in Old World camels based on newly annotated and reference-guided assemblies
Source: BMC Genomics. 2020 Sep 3;21:606. doi: 10.1186/s12864-020-06990-4 (PMC7468183; doi:10.1186/s12864-020-06990-4)
Supplement: Supplementary file 2 — Additional file 2: Supplemental Table 2. Means with 95% bootstrap confidence limits (CL, see Methods) of nucleotide diversity for alignments made with non-synonymous and synonymous SNPs and indels and only non-synonymous SNPs in HC (heavy-chain) immunoglobulin genes in DC (domestic camel), DROM (dromedary), and WC (wild camel). [file 12864_2020_6990_MOESM2_ESM.docx]

**Supplemental Table 2.** Means with 95 % bootstrap confidence limits (CL, see Methods) of nucleotide diversity for alignments made with non-synonymous and synonymous SNPs and indels and only non-synonymous SNPs in HC (heavy-chain) immunoglobulin genes in DC (domestic camel), DROM (dromedary), and WC (wild camel).

| **Non-synonymous SNPs** | **Mean** | **Upper 95% CL** | **Lower 95% CL** | **Species** |
| --- | --- | --- | --- | --- |
|  | 1.003664e-05 | 2.007329e-05 | -1.003664e-05 | DROM |
|  | 2.973608e-05 | 5.947216e-05 | -2.632341e-05 | DC |
|  | 1.080563e-04 | 2.036987e-04 | -1.674009e-05 | WC |
| **All SNPs and indels** | **Mean** | **Upper 95% CL** | **Lower 95% CL** | **Species** |
|  | 0.0004651567 | 0.0008144805 | -1.595646e-05 | DROM |
|  | 0.0005954219 | 0.0010261428 | -6.131850e-05 | DC |
|  | 0.0002285253 | 0.0003719989 | 5.000419e-05 | WC |
