# Supplementary figures and images for: Nucleotide diversity of functionally different groups of immune response genes in Old World camels based on newly annotated and reference-guided assemblies
Source: BMC Genomics. 2020 Sep 3;21:606. doi: 10.1186/s12864-020-06990-4 (PMC7468183; doi:10.1186/s12864-020-06990-4)

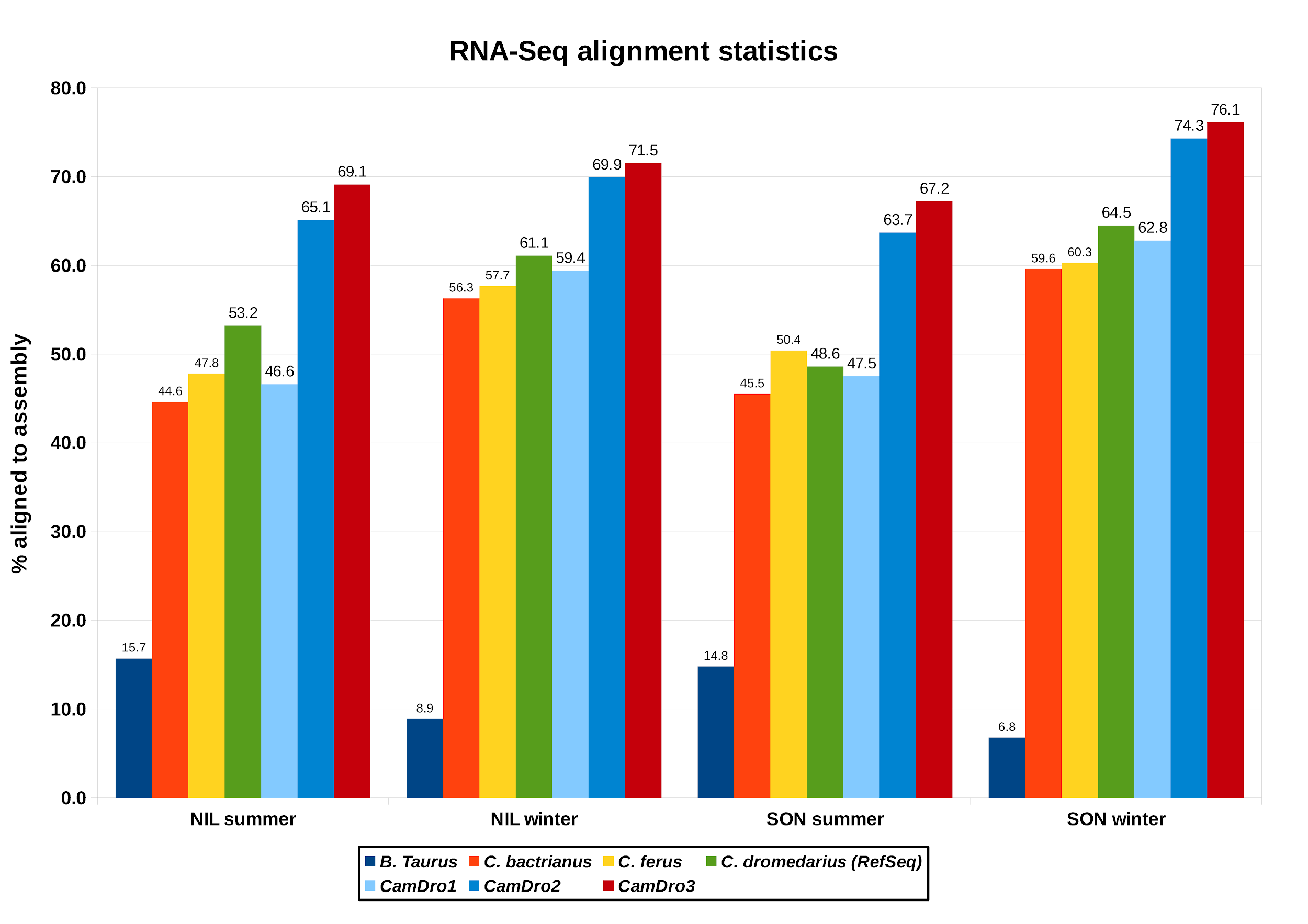


**Supplemental Figure 1.** RNA-Seq mapping rates.

Supplement: Supplementary file 3 — Additional file 3: Supplemental Figure 1. RNA-Seq mapping rates. [file 12864_2020_6990_MOESM3_ESM.docx]
