# Supplementary material for: Nucleotide diversity of functionally different groups of immune response genes in Old World camels based on newly annotated and reference-guided assemblies
Source: BMC Genomics. 2020 Sep 3;21:606. doi: 10.1186/s12864-020-06990-4 (PMC7468183; doi:10.1186/s12864-020-06990-4)
Supplement: Supplementary file 4 — Additional file 4: Supplemental Figure 2. D-GENIES (Cabanettes & Klopp, 2018) dot plot made with Minimap2 [68] whole-genome alignment between CamFer2 and the Camelus ferus genome (new-CamFer) assembly from Ming et al., [12]. Contigs are sorted and matches are filtered using the strong precision setting in D-GENIES. [file 12864_2020_6990_MOESM4_ESM.docx]

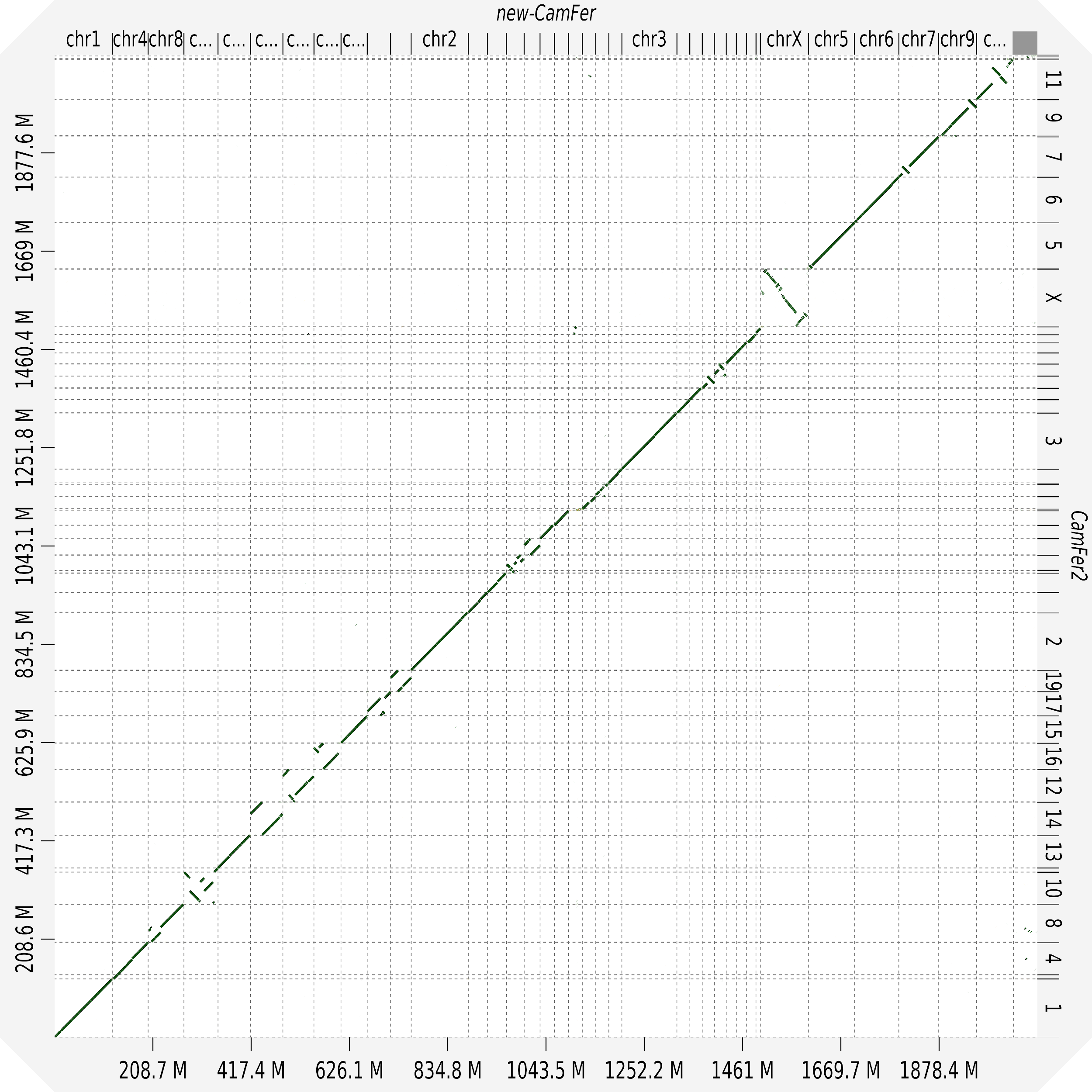


**Supplemental Figure 2**. D-GENIES (Cabanettes & Klopp, 2018) dot plot made with Minimap2 [67] whole-genome alignment between CamFer2 and the *Camelus ferus* genome (new-CamFer) assembly from Ming et al., [12]. Contigs are sorted and matches are filtered using the strong precision setting in D-GENIES.
