# Supplementary material for: Nucleotide diversity of functionally different groups of immune response genes in Old World camels based on newly annotated and reference-guided assemblies
Source: BMC Genomics. 2020 Sep 3;21:606. doi: 10.1186/s12864-020-06990-4 (PMC7468183; doi:10.1186/s12864-020-06990-4)
Supplement: Supplementary file 5 — Additional file 5: Supplemental Figure 3. Means with 95% bootstrap confidence intervals (see Methods) of nucleotide diversity for alignments made with non-synonymous and synonymous SNPs and indels (a) and only non-synonymous SNPs (b): MHC class I and II genes (top panel), innate (second panel), adaptive (third panel), and the rest of genome genes (bottom panel) for: DROM (dromedary, C. dromedarius), DC (domestic Bactrian camel, C. bactrianus), and WC (wild camel, C. ferus). Uppercase letters above upper 95% confidence limits indicate groups have different (non-matching letters) or not different (matching letters) means based on non-overlapping confidence intervals. [file 12864_2020_6990_MOESM5_ESM.docx]

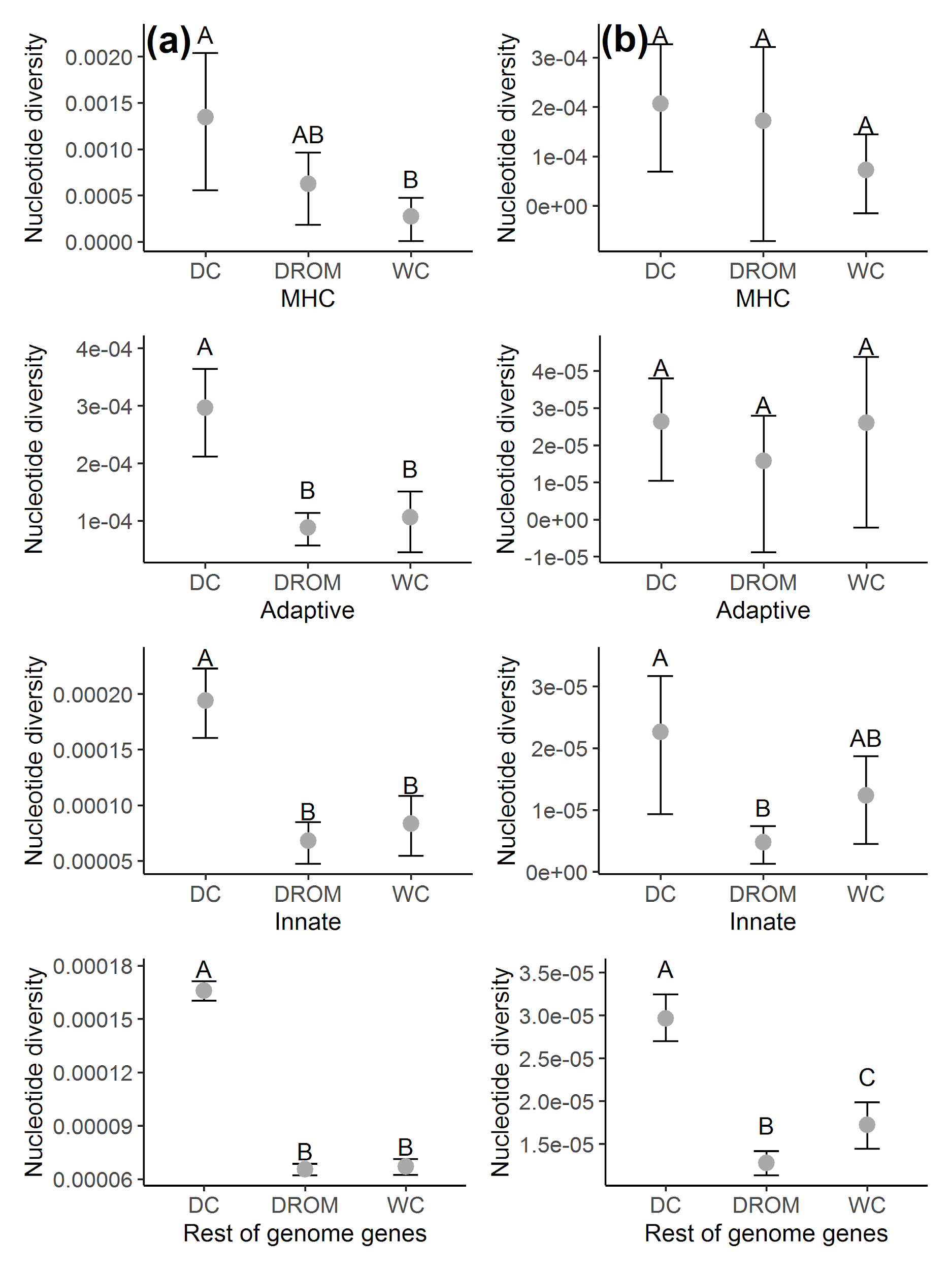


**Supplemental Figure 3.** Means with 95 % bootstrap confidence intervals (see Methods) of nucleotide diversity for alignments made with non-synonymous and synonymous SNPs and indels (a) and only non-synonymous SNPs (b): MHC class I and II genes (top panel), innate (second panel), adaptive (third panel) , and the rest of genome genes (bottom panel) for: DROM (dromedary, *C. dromedarius*), DC (domestic Bactrian camel, *C. bactrianus*), and WC (wild camel, *C. ferus*). Uppercase letters above upper 95 % confidence limits indicate groups have different (non-matching letters) or not different (matching letters) means based on non-overlapping confidence intervals.
