# Supplementary material for: Nucleotide diversity of functionally different groups of immune response genes in Old World camels based on newly annotated and reference-guided assemblies
Source: BMC Genomics. 2020 Sep 3;21:606. doi: 10.1186/s12864-020-06990-4 (PMC7468183; doi:10.1186/s12864-020-06990-4)
Supplement: Supplementary file 6 — Additional file 6: Supplemental Figure 4. Means with 95% bootstrap confidence intervals (see Methods) of nucleotide diversity for alignments made with (left) non-synonymous SNPs, (right) all SNPs and indels in HC (heavy-chain) antibody (immunoglobulin) genes in DC (domestic camel), DROM (dromedary), and WC (wild camel). Uppercase letters above upper 95% confidence limits indicate groups have different (non-matching letters) or not different (matching letters) means based on non-overlapping confidence intervals. [file 12864_2020_6990_MOESM6_ESM.docx]

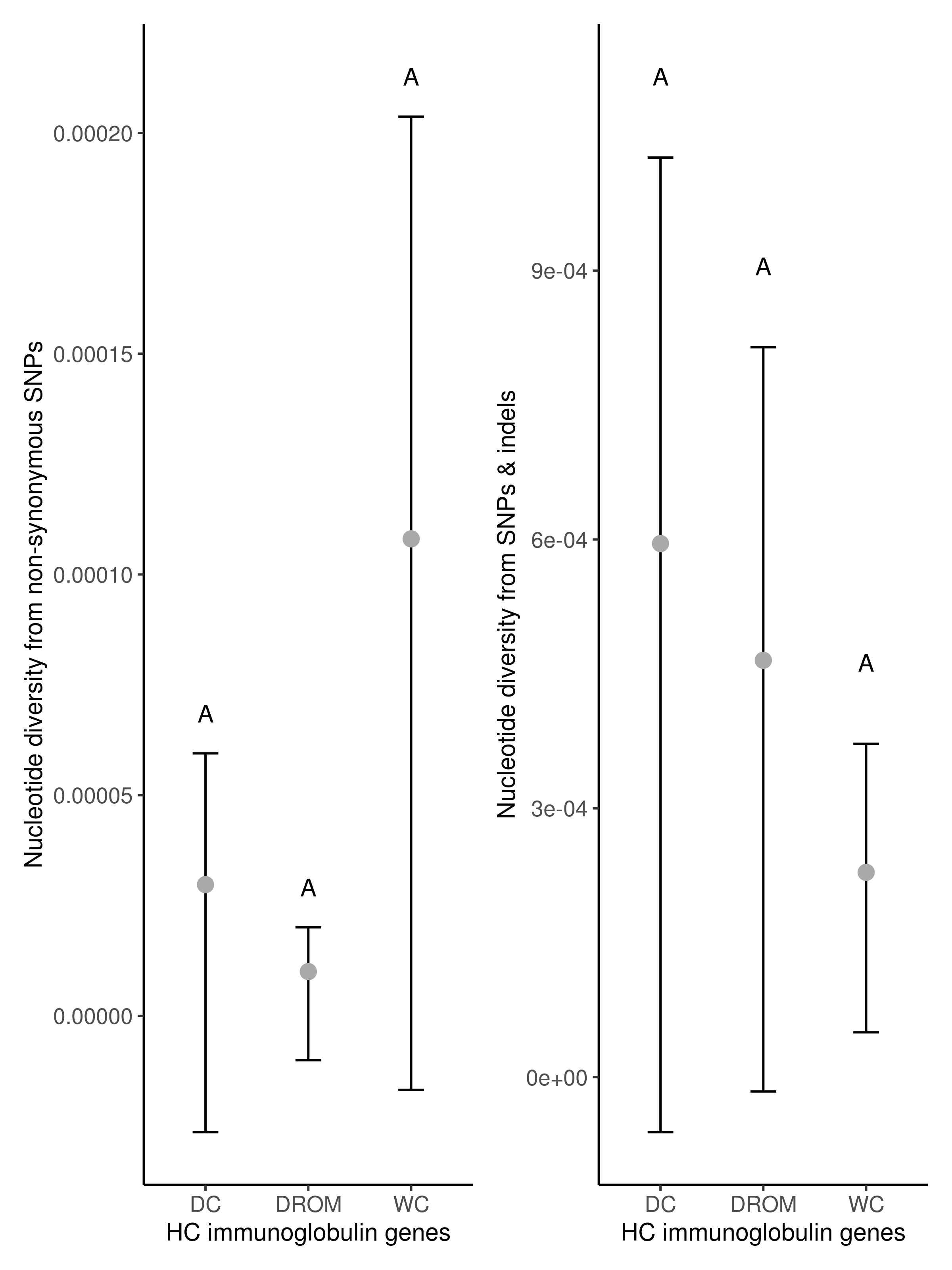


**Supplemental Figure 4.** Means with 95 % bootstrap confidence intervals (see Methods) of nucleotide diversity for alignments made with (left) non-synonymous SNPs, (right) all SNPs and indels in HC (heavy-chain) antibody (immunoglobulin) genes in DC (domestic camel), DROM (dromedary), and WC (wild camel). Uppercase letters above upper 95 % confidence limits indicate groups have different (non-matching letters) or not different (matching letters) means based on non-overlapping confidence intervals.
